# Evaluation of eye-related parameters and adverse events of rigid gas permeable contact lens and spectacles correction in infants with monocular aphakia after congenital cataract surgery: a retrospective clinical study

**DOI:** 10.1186/s12886-019-1088-z

**Published:** 2019-03-20

**Authors:** Junjue Chen, Ping Sun, Yan Wei, Xiaoli Kang

**Affiliations:** 1 Department of Ophthalmology in XinHua hospital, Shanghai Jiao Tong University School of Medicine, No.1665 Kongjiang road , Yangpu District, Shanghai, China; 2 Department of Ophthalmology, Eye & Ent hospital, Fudan University, Fenyang road, Xuhui District, Shanghai, China

**Keywords:** Rigid gas permeable contact lens, Congenital cataract, Unilateral, Aphkia, Spectacles, Persistent fetal vasculature

## Abstract

**Background:**

Congenital cataract is currently one of the leading blindness-causing eye diseases in children. Surgical treatment only opens the visual pathway for children. The postoperative recovery of visual function is also dependent on effective optical correction and visual function training. In this study, we analyzed the changes in eye-related parameters, adverse events and the annual cost of rigid gas permeable contact lens (RGPCL) and spectacles correction in infants with monocular aphakia after congenital cataract surgery.

**Methods:**

To analyze the postoperative visual acuity, strabismus, nystagmus, myopic shift, globe axial length growth, adverse events, patient adherence to patching, and annual cost for patients with unilateral congenital cataract who underwent cataract surgery. Rigid gas permeable contact lenses or spectacles were used to correct aphakia after congenital cataract.

**Results:**

Of the 49 patients, 20 patients with unilateral aphakia who used RGPCL were in group 1. Group 2 comprised 14 patients with persistent fetal vasculature (PFV) who used RGPCL, and there were 15 patients with spectacles in group 3. In group 1, there were important improvements in visual acuity, strabismus and nystagmus. In groups 2 and 3, there were no significant improvements in visual acuity, strabismus or nystagmus. Patients with a good adherence to patching had better visual acuity after the operation than patients who did not, in groups 1 and 3. There were no significant differences in myopic shift or rate of globe axial length growth among the 3 groups. No patients in group 1 had ocular disease that affected visual acuity. The mean annual expenses of the RGPCL group was 3965 yuan, and the mean annual cost of spectacles was 1140 yuan to 2500 yuan.

**Conclusion:**

RGPCL is a safe and effective optical correction method for patients with monocular aphakia after congenital cataract surgery. Spectacles are not an ideal optical correction. Using RGPCL to correct patients with PFV, the final visual acuity improved, but the difference was not statistically significant. There were no improvements in strabismus or nystagmus in patients with PFV.

## Background

Congenital cataract is currently one of the leading blindness-causing eye diseases in children. At present, most researchers suggest that cataract extraction surgery should be performed 6–8 weeks after birth to promote the development of the visual system [1]. Surgical treatment only opens the visual pathway for children. Postoperative recovery of visual function is also dependent on effective optical correction and visual function training. The Infant Aphakia Treatment Study (IATS) suggests that children less than 7 months old should not undergo primary intraocular lens (IOL) implantation; it is recommended for children 2 years of age or older [2]. The rapid and unpredictable growth of infant eyes makes it difficult to select the ideal IOL power to implant, and IOL implantation during early infancy has a higher risk of intraoperative and postoperative adverse events. Therefore, it is very important to choose a reasonable optical correction for children with early cataract surgery, especially for children with monocular cataract. A contact lens (CL) has become a useful optical correction method before IOL implantation, because of the effective reduction of the optical difference and aberration [3]. Compared with a soft CL, RGPCL has the advantages of high oxygen permeability, more adequate exchange of tears and no water content, thereby reducing the risk of infection. RGPCL also has a better imaging quality than a soft CL. In our study, we reported the ocular-related parameters, adverse events and annual cost of patients wearing either RGPCL or spectacles [4]. However, the use of contact lenses has not been popularized in China. Most parents choose spectacles for their children who are unable to have primary IOL implantation.

This retrospective study evaluated the postoperative outcomes in a series of patients who underwent cataract extraction within the sensitive period. We compared the ocular-related parameters of patients wearing a rigid gas permeable contact lens (RGPCL), patients wearing spectacles and patients with persistent fetal vasculature (PFV) wearing a RGPCL. The purpose of this study was to investigate which method of correction was the most useful way and to assess ocular-related parameters in patients with PFV wearing RGPCL.

## Methods

### Patients

Patients were selected from the Department of Ophthalmology in XinHua Hospital, Shanghai Jiao Tong University. Patients were diagnosed as having unilateral congenital cataract. All patients were treated between September 2008 and May 2017. This study was approved by the Ethics Committee of the XinHua Hospital, and conformed to the tenets of the Declaration of Helsinki. Each patient’s guardian or parents signed a consent form. The inclusion criteria were patients who received surgery for a visually significant congenital cataract (≥ 3 mm central opacity) in one eye, had their refractive error were corrected with RGPCL or spectacles, and had a follow-up period was longer than 6 months. Infants with a unilateral cataract due to PFV were allowed in the study. The exclusion criteria were an acquired cataract, a medical condition that might interfere with visual acuity testing, prematurity (< 36 gestational weeks), and glaucoma secondary to infantile cataract.

### Clinical examination procedures

We used a Snellen charts to measure visual acuity for children older than 3 years of age. For patients less than 3 years of age, the LEA Gratings test was used to evaluate molecular acuity. The Hirschberg test and prism cover test at both distance (5 m) and near (33 cm) fixation were used to determine whether patients had strabismus or nystagmus. Ocular examinations included cycloplegic refraction, intraocular pressure measurement (I-care), slit-lamp examinations, A/B-scan and an ultrasound tracings examination to measure the globe axial length and retina. For patients with RGPCL, the examination also included a measurement of corneal curvature, fluorescein and a blue light filter to check for any corneal or conjunctival staining, and the status of the RGPCL. Examinations of infant patients were conducted under chloral hydrate sedation.

### Calculation of myopic shift

The rate of change in refractive error was calculated as (D1 refractive error-D2 refractive error)/ (D1 month-D2 month). The D1 refractive error was the end refractive error of each stage, and the D2 refractive error was the initial refractive error of each stage. Furthermore, D1 month was the age corresponding to the D1 refractive error, and D2 month was the age corresponding to the D2 refractive error.

### Patching regimen

Starting the first postoperative week, parents were instructed to have their child wear an occlusive patch over the unoperated eye for 2 h/day for each month of age until the age of 12 months. From 12 months to 24 months, occlusive patching was prescribed for 3 h/day. Thereafter, patching was required for 6 h every day.

### Follow-up

Follow-up examinations were performed by the same investigator at 1 week, 1 month, and 3 months after cataract surgery. Subsequent follow-up examinations were conducted at 3–6 months intervals. Within a week after cataract surgery, patients underwent optical correction with a 2.0D overcorrection to provide a near point focus.

### Adherence to patching

Adherence to patching was assessed by asking the guardians or parents. Good adherence to patching was defined as reported patching at least 75% of the prescribed time. Three months after wearing RGPCL or spectacles, we evaluated patients’ visual acuity based on occlusion compliance.

### Statistical procedure

Statistical analyses were performed using SPSS software (19.0 IBM Corporation, Armonk, NY). First, the normal distribution of the data was tested. A paired t test or repeated measures ANOVA was used to compare the within-group differences. A Willcoxon signed-rank was used to compare differences among the treatment groups. Pearson’s Chi-squared test was used to compare the compliance rates between the groups of patients wearing RGPCL or spectacles. *P* < 0.05 indicated that the difference had statistical meaning.

## Results

### Clinical characteristics

There were 46 patients with monocular aphakia and monocular PFV enrolled in this study; 17 patients were female (39.5%). The median age of individuals was 3 years (ranging from 1 to 11 years). The patients were divided into 3 groups. There were 20 patients with RGPCL in group 1, 14 PFV patients with RGPCL in group 2, and 15 patients with spectacles in group 3. There were no PFV patients in group 3. The mean age at surgery 、mean length of follow-up period and final follow-up time are presented in Table 1. There were no significant differences in the follow-up period or the age at surgery among the 3 groups.

**Table 1:** Mean time of surgery, follow-up and final follow-up (months)

	Group 1	Group 2	Group 3
Age of surgery	8.45 ± 9.5	6.63 ± 4.57	10.34 ± 9.07
follow-up period(min-max)	24.55 ± 27.75(6–128)	30 ± 22.26(6–73)	39.82 ± 16.87(16–66)
Final follow-up	35.37 ± 29.42	37.69 ± 29.35	49.67 ± 20.34

### Visual acuity

Visual acuity increased with age in group 1, and there was an important improvement at final follow-up (*p* = 0.01). Compared to 24 months after surgery, visual acuity increased in group 3 at the final follow-up, but there was no significant difference. In the same age group, the visual acuity of group 1 was better than that of group 3 and there was a significant difference. The visual acuity of group 2 did not change significantly with age (Table 2).

**Table 2:** Visual acuity, myopic shift and rate of AL growth in 3 groups

Patient	Group1	Group2	Group3	P_1_
Visual acuity				
12 m	1.71 ± 0.14	2.18 ± 0.31	–	0.004
24 m	1.32 ± 0.11	2.03 ± 0.71	1.63 ± 0.24	0.03
36 m	0.96 ± 0.08	2.13 ± 0.61	1.46 ± 0.63	0.02
48 m	–	1.93 ± 0.83	1.57 ± 0.31	0.42
P_2_	0.01	0.44	0.65	
Myopic shift				
12 m	0.27 ± 0.24D	0.39 ± 0.30D	–	0.66
24 m	0.14 ± 0.24D	0.15 ± 0.08D	0.16 ± 0.15D	0.29
36 m	0.1 ± 0.09D	–	0.15 ± 0.12D	0.29
Rate of AL growth				
12 m	0.19 ± 0.12 mm	0.29 ± 0.13 mm	0.20 ± 0.11 mm	0.38
24 m	0.13 ± 0.13 mm	0.15 ± 0.14 mm	0.20 ± 0.11 mm	0.60
36 m	0.12 ± 0.08 mm	–	0.15 ± 0.10 mm	0.49

P_1_: *P* value of visual acuity between group 1 and group 3 at 24 months and 36 months after surgery; *P* value of visual acuity between group 1 and group 2 at 12 months after surgery; P value of visual acuity between group 2 and group 3 at 48 months after surgery. P_2_: *P* value among groups at different point; AL: axial length

### Eye alignment

Six patients (32%) in group 1 were orthotropic before cataract surgery and stayed orthotropic for the remainder of the follow-up. Two patients with exotropia and esotropia respectively became orthotropic in the subsequent follow-up period. There was a significant difference in eye alignment between the preoperative visit and final follow-up in group 1. Only 1 patient in group 2 was orthotropic before surgery and stayed orthotropic during follow-up. No patients with strabismus became orthotropic postoperatively. There was no significant difference in the angle of deviation in group 2. One patient in group 3 was orthotropic before surgery and stayed orthotropic for the remainder of follow-up. One patient with preoperative orthotropia had esotropia 42 months postoperatively. The degree of deviation at the final follow-up was greater than at the beginning, and there was a significant difference in group 3. Compared to group 3, the eye alignment in group 1 was significantly improved at the final follow-up (Table 3).

**Table 3:** Eye alignment and refractive error of 3 groups

Patient	Eye alignment			Refractive error		
	Pre-EA	Final-EA	P_1_	Pre-RE	Final-RE	P_2_
Group1	11.32 ± 9.68°	5.74 ± 5.52°	0.0002	20.24 ± 3.52D	17.14 ± 4.18D	< 0.0001
Group2	11.43 ± 5.56°	12.14 ± 6.36°	> 0.99	18.81 ± 3.61D	15.64 ± 4.34D	0.0039
Group3	8.64 ± 6.36°	13.18 ± 7.17°	0.02	18.50 ± 3.90D	15.13 ± 2.63D	0.0006
P_3_	0.42	0.004	–	0.42	0.36	

EA: eye alignment; RE: refractive error; P_1_: *P* value of eye alignment between pre- and final follow-up; P_2_: *P* value of refractive error between pre- and final follow-up; P_3_: *P* value of eye alignment between group 1 and group 3 at the same point; *P* value of refractive error among groups at the same point

### Nystagmus

The rate of nystagmus in group 1 was 63.2% before wearing RGPCL. At the final follow-up, there were 5 patients who had alleviation of nystagmus, and 2 patients with manifest nystagmus that became latent nystagmus. The remission rate of nystagmus was 58%. The rate of nystagmus in group 2 was 66.7% before wearing RGPCL. There was no improvement of nystagmus in group 2 at the final follow-up. The rate of nystagmus in group 3 was 58.3% preoperatively. There was no relief of nystagmus in group 3 at the end of the follow-up period.

### Adherence to patching

Six patients in group 1 had bad adherence to patching. Four patients in group 3 had no occlusion treatment in their home. Table 4 illustrates the visual acuity of these two types of patients in groups 1 and 3 and that all the patients in group 2 had occlusion treatment following the doctor’s advice. With good adherence to patching, there was a significant improvement in postoperative visual acuity in groups 1 and 3. With good adherence to patching, there was no significant difference in visual acuity between groups 1 and 3. Visual acuity had no statistical improvement in group 1 with bad adherence to patching. In group 3, with bad adherence to patching, postoperative visual acuity was worse than preoperative visual acuity, but there was no significant difference. With bad adherence to patching, the postoperative visual acuity of group 1 had a significant improvement over that of group 3.

**Table 4:** Visual acuity of good adherence and bad adherence to patching in 3 groups

patient	Good adherence to patching		P1	Bad adherence to patching		P2
	Pre-VA	Post-VA		Pre-VA	Post-VA	
Group 1	1.82 ± 0.47	0.96 ± 0.34	0.0005	1.71 ± 0.36	1.47 ± 0.18	0.13
Group 2	2.01 ± 0.25	1.52 ± 0.27	0.07	–	–	–
Group 3	1.35 ± 0.17	0.93 ± 0.10	0.007	1.99 ± 0.83	2.22(2.30–1.82)	0.73
P3	0.07	0.60		0.63	0.001	

P3: *P* value of visual acuity between group 1 and group 3

### Myopic shift

The mean refractive error of all 3 groups is presented in Table 3. The mean rate of change in the refractive error of each group is presented in Table 2. The myopic shift in group 2 was faster than that in group 1 within 12 months after birth. From 12 months to 24 months of age, the mean myopic shift in group 3 was the greatest among the 3 groups. From 24 months to 36 months of age, the mean myopic shift in group 3 was faster than that in group1. The data were not statistically significant between different groups.

### Globe axial length growth

Table 2 illustrates the trend of AL growth in the 3 groups from 12 months old to 36 months of age. The general trend of AL growth in the 3 groups was the same; all of them were slower with age. The rate of AL growth in group 2 was the fastest at the age of 12-months, and group 3 had the fastest rate at 24 months of age. There were no significant differences. The rate of AL growth in group 3 was faster than that in group 1 at 36 months of age, but there was no significant difference between them.

### Annual cost

The mean replacement rate of spectacles was 1.14 pairs/year. The mean annual cost of spectacles for children less than 2 years old was 2280 yuan, and the expense was 1140 yuan for children older than 2 years. If patients wore a press-on spherical lens, the yearly cost was 2500 yuan for patients less than 2 years old and 1710 yuan for children older than 2 years. The mean replacement rate of RGPCL was 1.56 pieces/year. The mean annual expenses of the RGPCL group was 3965 yuan. The annual cost included the contact lens, rewetting drops (Boston, changed every 3 months), and multiaction solution (Boston, changed every 1 month).

### Adverse events

In the RGPCL groups (including group 1 and group 2), 1 patient had a contact lens-associated complication (conjunctiva hyperemia), and no patients had ocular diseases that damaged visual acuity. In group 3, 3 patients developed disease-associated complications. Additionally, there were 3 patients who did not want to wear spectacles in group 3. There was no significant difference in the compliance between the two groups (RGPCL and spectacles, *P* = 0.193) (Table 5).

**Table 5:** Adverse events

Adverse event	RGPCL group	Group 3
Damage	6(17.6%)	8(53.3%)
Incompliance	4(11.7%)	4(26.7%)
Drop of RGPCL	8(23.5%)	–
Conjunctiva hyperemia	1(2.9%)	–
corectopia	–	2(13.3%)
Pupillary membranes	–	1(6.67%)

## Discussion

Many studies have reported the effectiveness and safety of a contact lens (CL) in correcting infantile aphakia [5, 6]. However, most of the patients enrolled in these studies were wearing a soft contact lens. Fewer studies have reported the results of wearing spectacles for children with monocular aphakia. Compared with a soft CL, RGPCL has high oxygen permeability. Furthermore, long-term RGPCL wear can better avoid corneal injury caused by hypoxia; it has a more adequate tear exchange, which can keep the corneal tissue clean in order to maintain a normal physiological metabolism; it does not contain water, so dust, bacteria and other substances will not be inhaled into the lens, greatly reducing the risk of infection; the imaging quality is high; and RGPCL has good formability and is not easy to deform.

Russell [7] et al. reported that the mean visual acuity of infants with monocular aphakia wearing CL was 0.81 LogMAR at 12 months old. Birch et al. reported that the mean visual acuity of 66 patients was 0.75 LogMAR at 12 months old, 0.55 LogMAR at 24 months old and 0.55 LogMAR at 36 months old. In our study, the mean visual acuity of the same age in group 1 was lower than the reports listed above. The possible reasons were that the operative time was relatively late and that some patients had a bad adherence to patching and visual function training. However, the visual acuity of patients in group 1 increased significantly with age. We believed that wearing RGPCL is an effective method that can improve the visual acuity of children with unilateral aphakia after cataract surgery. It has been reported that the best corrected visual acuity of patients with PFV was more than 1.3 LogMAR (20–71%) [8]. In our study, the final visual acuity in group 2 was 1.93 LogMAR. The visual acuity of group 2 and group 3 did not significantly increase with age. At the same time, the visual acuity of group 1 was significantly improved compared with that of group 3. The reason is that the spectacles caused a serious optical aberration and amplification effect, which leads to a serious disparity in the binocular image and the difficulty with fusion for the patients, making spectacles an unsatisfactory optical correction method.

Strabismus is more common in children with congenital cataract. Bothun [9] et al. reported that the incidence rate of strabismus was 64.6% at 12 months of age. Birch [10] et al. reported that the incidence rate of nystagmus was 71%. In our study, the incidence rate of strabismus and nystagmus in group 1 was 70 and 63.2%, respectively. Group 1 showed a significant improvement in strabismus and nystagmus at final follow-up. The eye alignment in group 3 had a statistical increase at final follow-up. Groups 2 and 3 had no statistical improvement in nystagmus. Congenital cataract and visual deprivation for over 6 weeks are considered to be risk factors for strabismus and nystagmus. Wearing RGPCL can make the retina imaging clear and reduce the difference between binocular imaging to 5–7% in patients with monocular aphakia. RGPCL can also promote the development of binocular vision and fusion function in children. In addition, wearing RGPCL can interfere with the movement of the eyeball by affecting the afferent information of the trigeminal nerve and reduce the intensity of nystagmus [11]. All this can effectively improve consciousness strabismus and nystagmus. In group 3, it is conceivable that the huge anisometropia caused by spectacles does not provide sufficient binocular input to the visual cortex, which therefore cannot yield simultaneous vision adequate to maintain the alignment of the two eyes.

Wilson [12] et al. reported that the axial growth rate of patients wearing CL within 12 months of age was 0.17 mm/month. In our study, the axial growth rate of group 1 within 12 months of age was 0.19 mm/month, and the rate of AL growth in group 2 was the fastest. After 12 months of age, the axial growth rate of group 3 was faster. In group 2, the greatest myopic shift within 12 months of age was due to the fastest axial growth rate. After 12 months of age, the faster myopic shift in group 3 was related to the rapid growth of the globe axis. The reason for the rapid increase of the globe axis within 12 months of age in group 2 is unknown.

In our report, there was a significant difference between the preoperative and postoperative visual acuity in patients who had good adherence to patching in groups 1 and 3. Previous reports suggested that achieving a good vision outcome for children with unilateral congenital cataracts requires early surgery, consistent optical correction and good adherence to patching of the unoperated eye [13].

Only one patient in the RGPCL group had a contact lens-associated complication, and no patients had an ocular disease that damaged visual acuity. The incompliance rate of the RGPCL group and spectacles group was 11.7 and 26.7%, respectively. Russell [14] et al. reported that only one patient wearing RGPCL had a related adverse event, which was a lens rupture. Due to RGPCL having a high permeability, small diameter, large lens activity and a certain degree of tear circulation during wear, it is difficult to cause corneal hypoxia and contact lens-related complications. However, because of the small diameter of the lens and steep corneal curvature of the patients, it is easy for RGPCL to be squeezed out of the palpebral fissure. For patients in group 3, the spectacles were large and the frame was pressed against the nose, causing the uncomfortable wearing for children.

Due to the retrospective study, some of our data were restricted. For example, although there was no statistical difference in the operation time between group 3 and group 1, the average operation time of group 3 was 2 months later than that of group 1; the VA missing data at age 12 months in the group 3; cataract density was not grouped preoperatively; different surgical techniques may be used during surgery; small number of patients who were enrolled, all of this can have an impact on our results. It has already been established that a CL provides a better optical correction in patients with monocular aphakia. In the future, we need to conduct a prospective study comparing RGPCL to a soft CL so that we can actually prove there are fewer complications and definite benefits to using RGPCL.

## Conclusion

In summary, RGPCL is a safe and effective optical correction method for patients with monocular aphakia after congenital cataract surgery. Spectacles are not an ideal optical correction. When RGPCL was used to correct patients with PFV, the final visual acuity improved, but the difference was not statistically significant. There was no improvement in strabismus or nystagmus in patients with PFV. There were no significant differences in myopic shift or the rate of globe axial length growth among the groups.

## Abbreviations

IOL: Intraocular lens
PFV: Persistent fetal vasculature
RGPCL: Rigid gas permeable contact lens
